# Through the Pharmacist’s Lens: A Qualitative Study of Antibiotic Misuse and Antimicrobial Resistance in Brazilian Communities

**DOI:** 10.3390/antibiotics14111074

**Published:** 2025-10-25

**Authors:** Timo J. Lajunen, Líria Souza Silva, Mark J. M. Sullman

**Affiliations:** 1 Department of Psychology, Norwegian University of Science and Technology, NO-7491 Trondheim, Norway; 2 Department of Social Sciences, School of Humanities and Social Sciences, University of Nicosia, Nicosia CY-1700, Cyprus; sullman.m@unic.ac.cy; 3 Department of Psychology, University of Helsinki, 00014 Helsinki, Finland; 4 Microorganisms Biotechnology Laboratory (LABIOM), Federal University of São João del-Rei (UFSJ), Divinópolis 35501-296, Minas Gerais, Brazil; liriasza@aluno.ufsj.edu.br

**Keywords:** antibiotics, community pharmacists, Brazil, AMR, reflective thematic analysis, self-medication

## Abstract

**Background**: AMR causes a large global health burden, with approximately 4.95 million deaths linked to bacterial AMR in 2019, 1.27 million due to AMR directly. Although Brazil mandated prescriptions for systemic antibiotics in 2010/2011, self-medication and access without prescriptions continue, with community pharmacists playing a vital part in antimicrobial stewardship (AMS). This study examined antibiotic misuse and AMR in Brazil through community pharmacists’ perspectives, emphasising their dual role as professional actors and frontline observers of public behaviour. **Methods**: We conducted 20 semi-structured interviews with community pharmacists and performed reflexive thematic analysis of their accounts, repeating five independent analytic cycles to confirm thematic robustness. **Results**: Six themes were consistently identified as recounted by pharmacists in their practice contexts: Access and Self-Medication; Relationships with Healthcare Professionals; Knowledge and Beliefs about Antibiotics; Use and Adherence; Healthcare System Barriers; and Regulation and Enforcement. Pharmacists mentioned regular requests for antibiotics without prescriptions, drug reuse, and significant impact from community, i.e., from relatives, and peers. The common misunderstanding was that antibiotics treat viral illnesses. Structural issues, for instance GP appointment costs and long waits, made patients seek help from pharmacies. Due to regulation being applied inconsistently, pharmacies struggled to refuse unsuitable requests. **Conclusions**: Framed through pharmacists’ dual vantage as professionals and frontline observers, the findings highlight intertwined factors underpinning inappropriate antibiotic use in Brazil and support a multi-pronged intervention spanning health system strengthening, professional education, economic considerations, and community engagement.

## 1. Introduction

Antimicrobial resistance (AMR) is compromising clinical care routines and pressuring health systems and economies globally. In 2019, approximately 4.95 million deaths were linked to bacterial AMR, with 1.27 million directly attributed to resistant infections [1,2,3]. AMR affects a wide range of interventions, including chemotherapy, surgical procedures, and obstetric care, by increasing the likelihood of hard-to-treat infections, lengthening hospital admissions, and raising healthcare costs. The World Health Organization has advocated for rapid, coordinated “One Health” action involving human, animal, and environmental sectors, though progress by national governments varies [4].

Inappropriate use of antibiotics, particularly self-medication without professional input, is a major driver of bacterial AMR. Such behaviour is frequent in numerous low- and middle-income countries (LMICs), where limited access to primary care and diagnostics encourages pharmacy-first health-seeking. In Brazil, these challenges are especially pronounced due to its patchwork of public and private healthcare and significant regional disparities in access. Brazil has required prescriptions for systemic antibiotics since late 2010 and has introduced further regulatory updates upholding prescription and documentation requirements, but non-prescription use remains common [5,6,7]. Interrupted time-series analyses confirm that the 2010/2011 regulations reduced population-level antibiotic consumption, but long-term effectiveness has been limited by inconsistent enforcement and substitution with other medicines [7,8].

Community pharmacists in Brazil occupy a visible, trusted position at the front line of healthcare access, often acting as first contact for patients with minor ailments. In under-resourced areas, they may serve as de facto primary care providers [9]. Their responsibilities include triaging and supporting patients, offering education and guidance on correct medicine use, managing minor illnesses, and reducing risks of self-medication. However, community pharmacies in Brazil face obstacles such as lack of standardisation, inadequate remuneration models, regulatory and competency ambiguity, and limited stakeholder communication [9,10]. Some studies report suboptimal dispensing by pharmacists, characterised by insufficient patient assessment, limited advice, and poor record-keeping, with antimicrobial agents sometimes dispensed without adequate checks or prescriptions, thereby impeding stewardship and perpetuating inappropriate use [10,11].

Self-medication with antibiotics, reported by Tiguman et al. (2020), increased in adults in Manaus, in the Brazilian Amazon, from 19.2% in 2015 to 30.7% in 2019 [12]. National retail data reveal significant regional variation and continuity in policy, including the 2021 regulatory update that sustained prescription control, suggesting that legal mechanisms alone are insufficient without inspection, feedback, and public communication [5]. National and international studies consistently underscore persistent public misconceptions about antibiotics, such as their use for colds or influenza or discontinuing them once symptoms improve, which increase demand and complicate pharmacist-patient interactions [13].

Targeting pharmacy practice is therefore crucial. Evidence from LMICs shows that combining regulation with pharmacist training and public education reduces non-prescription sales [14]. Point-of-care C-reactive protein testing alongside staff training and referral pathways has been piloted in community pharmacies, reducing unnecessary antibiotic provision [15,16,17]. Communication tools like pictogram-based dosing instructions also improve understanding and adherence.

Understanding community pharmacists’ experience in dispensing antibiotics and addressing AMR is vital before developing new interventions. This qualitative study examines antibiotic misuse and AMR in Brazil through the perspectives of community pharmacists, foregrounding their dual role as professional actors and frontline observers of public behaviour. We used reflexive thematic analysis of interviews to explore (i) pharmacists’ professional experiences and challenges within routine practice, (ii) their frontline observational accounts of community antibiotic use and its drivers, and (iii) feasible intervention targets at pharmacy and system levels. The study contributes by making explicit the interpretive lens of pharmacists’ accounts that bridge professional practice and community behaviour.

## 2. Methods

### 2.1. Study Design

This study employed a qualitative research design using thematic analysis to explore community pharmacists’ perspectives on antibiotic self-medication and antimicrobial resistance. The study was conducted in accordance with the Consolidated Criteria for Reporting Qualitative Research (COREQ) guidelines [18]. Pharmacists’ accounts were the unit of analysis, encompassing both professional experiences and frontline observations of public behaviour; no direct patient or physician data were collected.

### 2.2. Participants and Setting

Community pharmacists practising in Brazil were recruited through purposive sampling to ensure diverse representation across different regions, pharmacy types, and years of experience. Inclusion criteria included: (1) licensed pharmacists working in community pharmacy, (2) a minimum of one year of practice experience, and (3) willingness to participate in audio-recorded interviews. Exclusion criteria included hospital-based pharmacists and those working exclusively in administrative roles.

A total of 20 community pharmacists participated in the study. The sample had a mean age of 35.2 years (range: 26–46), included 12 women, and all participants were the technically responsible pharmacists for their establishments. Participants were drawn from three of Brazil’s five major regions, with the majority (n = 15) practising in the Southeast in cities across Minas Gerais, São Paulo, and Rio de Janeiro. One participant was from the North region (Manaus, Amazonas) and one from the Northeast (Natal, Rio Grande do Norte).

### 2.3. Data Collection

Semi-structured interviews were conducted using a topic guide developed based on a literature review and expert consultation. The interview guide covered such topics as antibiotic dispensing practices, patient interactions, barriers to appropriate prescribing, AMR awareness, and suggestions for improvement. Interviews were conducted in Portuguese, lasted 45–90 min, and were audio-recorded with participant consent.

### 2.4. Data Analysis

The qualitative interview data were transcribed and translated from Portuguese to English using the AI-powered software Avidnote (Mar 14 version) [19]. To ensure linguistic and contextual accuracy, all automated outputs were manually reviewed and corrected by the second author, who is fully bilingual. This hybrid approach combined efficiency with the preservation of data integrity and nuance.

Thematic analysis was conducted following Braun and Clarke’s reflexive methodology [20,21]. Reflexive thematic analysis is one of the most widely used approaches in the social and health sciences, particularly for exploring professional practices and lived experiences. Consistent with reflexive thematic analysis, themes were treated as patterns of meaning actively constructed by the researchers, emphasising reflexivity and transparency rather than inter-coder reliability. In this study, themes describe how pharmacists make sense of their practice environments and their observations of community antibiotic use, and they are not intended to estimate behavioural prevalence. The process involved six iterative phases: (1) data familiarisation, (2) code generation, (3) theme searching, (4) theme reviewing, (5) theme naming/defining, and (6) report production. The analysis was repeated five times to ensure the robustness of the identified themes.

### 2.5. Ethical Considerations

Ethical approval was obtained from the relevant institutional review board (SSERB 390; 7 August 2024). All participants provided written informed consent, and confidentiality was maintained throughout the study. Participants were assigned pseudonyms, and identifying information was removed from transcripts.

## 3. Results

Analysis of the 20 Portuguese-transliterated interviews identified six themes that consistently emerged across five independent runs of reflexive thematic analysis. The themes include descriptions of patient behaviours and lay beliefs as reported by pharmacists; these are presented to characterise pharmacists’ practice environment and observational insights, not to claim direct patient evidence. Table 1 summarises each theme, including the number of unique interviewees who mentioned it, the total number of coded instances across the dataset, and a concise description. The frequent occurrence of specific themes—mainly Healthcare System Barriers and Access and Self-Medication—emphasises how crucial they are in influencing antibiotic use and dispensing habits. Even the least frequent theme, Regulation and Enforcement, appeared in 13 interviews, highlighting its relevance.

### 3.1. Theme 1: Access and Self-Medication

From the pharmacists’ perspective, these accounts reflect frontline experiences of requests and self-medication practices encountered at the counter. This theme emerged as participants consistently described the widespread attempts to obtain antibiotics without medical consultation. Accounts revealed a recurrent pattern in which individuals approached pharmacies directly, requesting antibiotics at the counter as if they were routine, over-the-counter medications. Alongside these requests, participants emphasized the common practice of reusing leftover antibiotics from previous treatments. In such cases, customers arrived with clear ideas about which medication they wanted, often guided not by professional advice but by past experience or the recommendations of friends and family. Together, these behaviours reflect a strong and persistent culture of self-medication that appeared across settings, but with particularly sharp contours in underserved areas where barriers to accessing formal healthcare were most pronounced. The following two extracts illustrate how this theme was expressed in practice:

“Many clients come directly to the counter asking for antibiotics without a prescription, often citing past experience or advice from friends or relatives. Pharmacists frequently need to explain the requirement for a medical evaluation before dispensing.”

(Participant 7)

“Some customers insist they can manage their condition because they have used the same antibiotic before, keeping leftover doses from previous treatments to avoid a doctor’s visit.”

(Participant 12)

These extracts show how perceived familiarity with antibiotics fosters a sense of confidence in their use, with individuals treating antibiotics as tools they can manage independently, bypassing medical pathways altogether. The insistence on reusing familiar drugs highlights how patients rely on their own judgment or community knowledge, rather than seeking clinical evaluation. In several accounts, pharmacists positioned these practices as pragmatic responses to healthcare gaps: when services were scarce, costly, or slow, patients relied on the most immediately available option. In rural contexts especially, where healthcare facilities were distant or appointments hard to secure, self-medication was described not only as common but as an almost necessary adaptation to structural constraints.

### 3.2. Theme 2: Relationship with Healthcare Professionals

Analysis revealed complex dynamics in the pharmacist–patient–physician relationship. Pharmacists frequently positioned themselves as intermediaries—redirecting patients to doctors when antibiotic use was questionable, while also managing the tension between professional boundaries and customer expectations.

“Pharmacists often act as gatekeepers, redirecting patients to doctors for proper diagnosis when antibiotic use was questionable.”

(Participant 3)

“Tensions arise when customers expect pharmacists to diagnose and prescribe, which is beyond their professional role.”

(Participant 10)

“Here…people often go directly to the pharmacy when they cannot get adequate medical appointments, so they end up acquiring antibiotics without prior medical evaluation.”

(Participant 1)

These accounts demonstrate how pharmacists negotiate dual responsibilities: protecting public health by ensuring appropriate antibiotic use, and maintaining trust with clients who may view them as accessible substitutes for physicians.

### 3.3. Theme 3: Knowledge and Beliefs About Antibiotics

Pharmacists report these community-held understandings as they arise in consultations, shaping demand and counselling needs in practice. Participants consistently reported that misconceptions about antibiotics were widespread and deeply rooted in everyday practices. Lay beliefs, often shared within families, neighbourhoods, or informal advice networks, strongly shaped how individuals requested and used antibiotics. These beliefs gave antibiotics a kind of “universal medicine” status, which made them appear useful for a wide range of conditions, including those where they were neither needed nor effective.

One of the most striking misconceptions was the idea that antibiotics could treat viral illnesses. As one participant observed:

“Patients often believe antibiotics can cure viral infections like the common cold or flu.”

(Participant 15)

This conviction was echoed repeatedly, suggesting that antibiotics are perceived less as targeted treatments and more as general remedies. The repetition of this belief across interviews reinforced its persistence:

“Many patients believe antibiotics can be used for any illness, even viral infections like flu or colds.”

(Participant 3)

The extracts illustrate how antibiotics are treated as catch-all solutions, blurring the crucial distinction between bacterial and viral illnesses. Pharmacists described these requests as common, and often had to explain that antibiotics would not help with flu-like symptoms.

Another recurrent lay logic concerned the relationship between symptom severity and the need for antibiotics. Instead of understanding antibiotics as linked to specific types of infection, many customers evaluated their necessity according to how ill they felt:

“Some customers judge the need for antibiotics based solely on the severity of their symptoms rather than the underlying cause.”

(Participant 4)

This shows that patients were guided by subjective impressions of illness rather than medical criteria, using antibiotics almost as reassurance when symptoms seemed “serious enough.” Such perceptions created additional pressure for pharmacists, who had to balance patient expectations with professional standards. Even when pharmacists attempted to correct misconceptions, some patients continued to hold onto incorrect beliefs. As one explained:

“Even after professional guidance, people sometimes keep the wrong idea that antibiotics cure everything.”

(Participant 10)

This highlights the durability of these misconceptions: once embedded in personal or community experience, they were resistant to change, even when challenged with accurate information.

The circulation of community-based knowledge further reinforced misunderstandings. Information shared among family, friends, or neighbours could carry strong authority, even when it was inaccurate:

“Information circulates in the community that is not always correct, and patients repeat it with conviction.”

(Participant 12)

This extract illustrates how misinformation was not just an individual error but part of a wider social process, in which incorrect advice was repeated and legitimised through collective confidence. Pharmacists, therefore faced not only individual misunderstandings but also entrenched cultural patterns that spread and maintained misconceptions.

Taken together, these extracts underline the challenge of combating misinformation. Pharmacists frequently emphasised their role in clarifying misunderstandings, explaining the differences between bacterial and viral illnesses, and reinforcing appropriate antibiotic use. Yet the persistence of lay beliefs demonstrated the limits of these efforts, showing how strongly community narratives shaped patient behaviour.

### 3.4. Theme 4: Use and Adherence to Treatment

Descriptions of use and adherence below are pharmacists’ observational accounts that inform their counselling and stewardship actions. Once antibiotics were obtained, participants described highly variable patterns of adherence. Pharmacists reported that inappropriate use was not confined to obtaining antibiotics without prescriptions; it also extended into the ways patients consumed and managed the medications once they had them. These behaviours often undermined the effectiveness of treatment and increased the risk of resistance. Several recurring practices were identified, ranging from stopping treatment early to altering dosage schedules or redistributing pills among household members.

A very common pattern was the premature interruption of treatment once symptoms improved. As one participant explained:

“Many patients stop taking antibiotics as soon as they feel better, without completing the treatment.”

(Participant 2)

Another emphasised how this tendency was widespread and had long-term risks:

“Customers sometimes stop taking antibiotics when they feel better, leading to incomplete courses and risk of resistance.”

(Participant 6)

These observations underline how patients often prioritised immediate relief over the full course of treatment. The sense of improvement was interpreted as recovery, leading to the belief that continued antibiotic use was unnecessary. Pharmacists highlighted that such behaviours directly undermined treatment effectiveness and contributed to the persistence of infection.

Another set of practices involved altering the dosage or dividing medication among others. One pharmacist noted:

“Some patients adjust doses on their own to ‘stretch’ the medication supply or share it with others.”

(Participant 14)

This behaviour suggests both financial constraints and a perception of antibiotics as flexible resources that could be managed outside medical guidance. Sharing antibiotics within households reflected the idea of communal medicine use, where pills were not tied to a single prescription but to a shared notion of treatment.

Dose reduction was another manifestation of self-management. One participant observed:

“There are patients who cut tablets in half to reduce what they consider to be excessive doses.”

(Participant 11)

This practice was often described as an attempt to avoid perceived risks or side effects, but it further compromised therapeutic effectiveness. Patients substituted personal judgment for medical instruction, demonstrating a lack of trust in prescribed dosing.

Side effects were also a major factor influencing adherence. As one pharmacist explained:

“Side effects, like stomach upset, make people interrupt the treatment before finishing it.”

(Participant 8)

Here, the discomfort associated with treatment often outweighed patients’ motivation to continue. Even when pharmacists explained that such reactions were temporary, many individuals considered them sufficient reason to abandon the course.

Pharmacists consistently emphasized how these practices reflected not only misunderstanding but also a tendency to adapt treatments to personal logic or convenience:

“It is common for customers to adapt the treatment to their own logic, not to medical recommendations.”

(Participant 13)

This extract captures the essence of the theme: antibiotic use was frequently reshaped according to subjective reasoning, practical constraints, or experiential knowledge rather than clinical guidance.

Taken together, these patterns show that issues of use and adherence extend well beyond access to antibiotics. They highlight the tension between prescribed medical regimens and patient-driven practices, in which treatment was modified, shortened, or redistributed. Pharmacists often attempted to counteract these risks by giving clear instructions, writing schedules directly on medication packages, or offering verbal reinforcement. Nevertheless, patient adherence remained inconsistent, reflecting the challenge of aligning professional recommendations with everyday practices and beliefs.

### 3.5. Theme 5: Healthcare System Barriers

Participants repeatedly emphasised that structural limitations within the healthcare system were central drivers of inappropriate antibiotic use. These barriers created conditions in which pharmacies were not just convenient alternatives but often the only accessible point of care. Accounts highlighted long waiting times in public services, high costs in private clinics, geographical distance from healthcare facilities, and limited availability of medical professionals. Each of these factors converged to encourage patients to bypass formal medical consultations and turn directly to pharmacists when seeking antibiotics.

One of the most frequently cited problems was the excessive waiting time in public clinics. Patients often weighed the delay against the immediate need for relief and opted for pharmacies instead:

“Long wait times at public health clinics lead some patients to seek antibiotics directly from pharmacies.”

(Participant 1)

This extract illustrates how patients interpreted waiting not merely as an inconvenience but as an obstacle to care. The expectation of long queues meant that the pharmacy became the faster and more efficient pathway, even if it bypassed professional diagnosis.

Another important barrier was financial. The cost of private consultations was described as beyond the reach of many families, making pharmacies the only feasible alternative. As one participant noted:

“Consultation costs in private clinics are prohibitive for lower-income patients, pushing them toward self-medication.”

(Participant 11)

A similar point was expressed in another interview:

“Private consultations are very expensive, so for low-income people the pharmacy becomes the first option.”

(Participant 4)

Together, these comments underline how socioeconomic constraints shaped health-seeking behaviours. Rather than being a matter of preference, bypassing doctors was often an economic necessity.

Time constraints also played a role in discouraging formal care. For families already stretched by work obligations, attending long consultations was simply not practical:

“Many families cannot afford to take time off work for long clinic appointments, so they try to solve directly with us.”

(Participant 14)

This extract shows how systemic barriers extended beyond financial costs into the realm of everyday life, where time itself was a scarce resource.

Geographical accessibility was another recurrent issue, especially in rural or remote areas. In such contexts, pharmacies represented the only realistic option for medical treatment:

“In rural areas, the health post may be far away, and the pharmacy is the only practical access.”

(Participant 6)

Here, distance functioned as a barrier just as powerful as cost or waiting times. For many, the pharmacy filled the void left by inaccessible or under-resourced health facilities.

Limited availability of medical professionals further compounded these barriers. Certain periods, such as weekends or holidays, intensified patient reliance on pharmacies:

“On weekends and holidays, when doctors are not available, many requests for antibiotics appear.”

(Participant 8)

This quote illustrates how gaps in service provision created peaks of demand, making pharmacies the only contact point during these times. Beyond this, a more general lack of medical staff was noted:

“The system does not provide enough doctors, and this creates pressure on pharmacies to meet demands.”

(Participant 7)

These comments highlight the structural shortage of human resources in healthcare, placing an added burden on pharmacists who were approached to fill the gap.

Taken together, these accounts demonstrate how systemic limitations in the healthcare system—long waits, high costs, geographic barriers, and insufficient staff—consistently redirected patients toward pharmacies as a primary source of treatment. Rather than being isolated cases, these were recurring patterns across different contexts. In practice, pharmacists were positioned as the first, and sometimes only, point of contact for patients, revealing how deeply structural barriers fuelled the inappropriate use of antibiotics.

### 3.6. Theme 6: Regulation and Enforcement

The final theme captured how formal regulations and their enforcement shaped antibiotic dispensing practices. Across interviews, pharmacists described relying on the prescription requirement as both a protective measure and a tool for managing customer expectations. However, the strength of this safeguard varied depending on the consistency of enforcement in their local contexts. For some, strict application of the law provided external legitimacy for refusal, while for others, weak or inconsistent enforcement left them vulnerable to patient pressure and competing practices from less regulated establishments.

Pharmacists often positioned the prescription rule as a clear professional boundary, one that allowed them to refuse inappropriate requests without appearing arbitrary. As one explained:

“We rely on the prescription rule to say no to patients who want antibiotics without one.”

(Participant 2)

By invoking the regulation, pharmacists shifted the responsibility from themselves to the legal framework, using the rule as an external authority that justified their actions. Another participant described this directly:

“Pharmacists use the law as an argument, to show it is not personal but obligatory.”

(Participant 9)

These extracts illustrate how regulations provided pharmacists with a shield against patient dissatisfaction. The law served not only as a formal requirement but also as a communication tool for explaining refusals.

Yet enforcement was not uniform, and pharmacists working in areas with weaker oversight reported facing greater challenges. As one stated:

“In regions with weak enforcement, pharmacists face more frequent challenges from customers expecting antibiotics without prescriptions.”

(Participant 9)

This reflects how patient expectations were shaped by local practices: in contexts where regulations were inconsistently applied, patients assumed that antibiotics could still be obtained without prescriptions, and often pressured pharmacists to comply. Another participant noted a similar problem:

“Where enforcement is weak, patients expect to get antibiotics easily and pressure us more.”

(Participant 7)

These accounts highlight how uneven enforcement undermined the effectiveness of regulation, placing additional strain on pharmacists to hold their ground against persistent demands.

In some cases, the sale of antibiotics without prescriptions continued, depending on the strength of local controls:

“The sale without prescription still happens in some pharmacies, depending on how strong the control is.”

(Participant 12)

This extract illustrates how variability in enforcement not only influenced patient expectations but also created competition between pharmacies, with some adhering strictly to the rules and others allowing exceptions. Such inconsistency weakened the overall impact of regulation by teaching patients where they could bypass restrictions.

As one participant summarised, enforcement itself was not always reliable:

“Enforcement helps, but it is inconsistent, so patients learn where they can get antibiotics without problems.”

(Participant 19)

This highlights how regulations, while effective on paper, often failed to operate uniformly in practice. Patients developed strategies to navigate the uneven regulatory landscape, shifting between pharmacies until they found one willing to comply with their requests.

Taken together, these accounts show how regulatory frameworks both empowered and constrained pharmacists. Where enforcement was strong, pharmacists relied on the law as an authoritative safeguard that legitimised refusal and maintained professional boundaries. Where enforcement was weak or inconsistent, however, pharmacists were left more vulnerable to pressure, and patients quickly learned to exploit variability across establishments. Thus, regulation functioned as both a critical protective tool and a source of tension, its impact determined less by the rules themselves than by the strength of their enforcement.

Across the dataset, six interrelated themes shed light on the complex factors driving antibiotic use and dispensing. Self-medication and misconceptions about antibiotics were widespread, reinforced by systemic healthcare barriers and inconsistent regulation. Pharmacists frequently occupied a mediating role, balancing professional responsibility with public expectations, while struggling to promote adherence once antibiotics were obtained. Together, these findings illustrate the interplay of individual behaviour, professional practice, and systemic structures in shaping antibiotic use.

## 4. Discussion

This qualitative study identified six interrelated themes through community pharmacists’ perspectives, combining their professional experiences with their frontline observations of community behaviour: Access and Self-Medication, Relationships with Healthcare Professionals, Knowledge and Beliefs about Antibiotics, Use and Adherence to Treatment, Healthcare System Barriers, and Regulation and Enforcement. Taken together, these findings underscore the persistent non-prescription use of antibiotics, driven by barriers to healthcare access, widespread misconceptions, and inconsistent regulatory frameworks. Pharmacists frequently serve as the initial point of contact, mediating public expectations against professional and ethical standards. This is consistent with the study’s framing through pharmacists’ professional and observational accounts.

Our findings are in line with national and regional evidence that self-medication—including antibiotic self-medication—remains common in Brazil and Latin America, and that pharmacies are often the first point of contact for minor ailments [9,22,23]. Qualitative work from Colombia similarly documents the social and system drivers (e.g., time/cost barriers, trust in pharmacy staff) that normalise non-prescription antibiotic use, underlining the cross-context relevance of our themes [24]. Investigations also show that legal restrictions on over-the-counter antibiotic sales reduce inappropriate access only when accompanied by multifaceted enforcement and communication strategies [14]. Evidence from the Mexico–U.S. border further illustrates the role of weak control and cross-border access in sustaining inappropriate use, underscoring that policy must be matched with implementation capacity [25].

According to the participants’ accounts, a rational process connects structural limitations to self-medication: factors like lengthy waiting times, limited appointment availability, various costs, and geographical distance prompt individuals to seek care at pharmacies, where they utilise communal knowledge (e.g., prior usage, recommendations from peers) to acquire or repurpose antibiotics. At the same time, long-held lay beliefs are observed (e.g., antibiotics being effective in the treatment of viral diseases). In the Brazilian context, this aligns with evidence that, despite ANVISA’s prescription requirements, enforcement and practice vary, and some pharmacies have historically circumvented rules [14,26,27]. Recent Latin American surveys show that public and professional pressures frequently shape antibiotic decisions; for example, healthcare workers across five countries reported limited access to local guidelines and substantial patient/family pressure to prescribe, which likely complicates community-facing stewardship [28].

At the practice level, the prominence of Use and Adherence in pharmacists’ accounts implies that stewardship should not stop at controlling access. Structured pharmacist-delivered counselling, simple written dosing schedules, and low-literacy communication aids (e.g., pictograms) can improve comprehension and short-course adherence [29,30,31]. A Brazilian study shows that generic, imported pictograms may be poorly understood by older adults unless culturally adapted, arguing for locally designed visuals embedded in counselling [32].

At the system level, research indicates that augmenting pharmacist triage with point-of-care testing (e.g., C-reactive protein, CRP) can safely reduce unnecessary antibiotic supply for respiratory tract infections: a cluster RCT in Nigeria reduced non-prescription antibiotic dispensing, and a Northern Ireland pilot showed feasibility in community pharmacies [16]. A recent LMIC review indicates that POCT can be successfully implemented in private pharmacy and drug retail settings, provided that protocols, training, and referral pathways are in place [15]. Policy levers should therefore combine consistent prescription-only enforcement with supportive tools (diagnostics, standard counselling protocols), professional training, and public communication that targets dominant misconceptions [14].

The salience of misinformation in interviews aligns with multi-country surveys (including Mexico) documenting widespread misunderstandings—e.g., that antibiotics work for colds/flu or that the body, rather than bacteria, becomes resistant—and underscores the need for sustained public messaging embedded in pharmacy encounters [4]. Complementary Latin American evidence links self-medication to access barriers and perceived effectiveness, highlighting targets for micro-interventions at the counter [24,33]. Brief myth-busting, clear “no-antibiotic” return/referral criteria and culturally adapted educational materials could be high-reach channels in Brazil, where pharmacies are highly accessible [33].

### 4.1. Strengths and Limitations

Strengths of this study include a reflexive thematic approach with convergence across five independent analytic runs and coherent triangulation across participants, enhancing credibility and dependability [20,21]. Limitations include potential social desirability bias (pharmacists portraying gatekeeping positively), the absence of physician/patient data for triangulation, and the study’s focus on Portuguese-transliterated interviews from a single national context, which may limit transferability to other LMIC settings with different regulatory and health-system arrangements. The findings reflect pharmacists’ perspectives on their practice environment and their observations of public behaviour; consequently, patient and physician viewpoints are indirectly represented through pharmacists’ accounts.

### 4.2. Future Directions

Mixed-methods evaluations should test pharmacy-anchored stewardship bundles that integrate (i) standardised refusal scripts and pictogram-enhanced dosing aids; (ii) targeted myth-rebuttal materials aligned with WHO messaging; (iii) CRP POCT with clear referral thresholds; and (iv) local enforcement audits coupled with feedback to pharmacies. Interrupted time-series of antibiotic sales and simulated-client studies can quantify impact and detect displacement effects (e.g., shifts to prescribed channels), while qualitative follow-ups can map acceptability and equity implications. Regional work should attend to contextual variation—e.g., guideline access and patient pressure observed among Latin American healthcare workers—to tailor feasible, scalable interventions [28].

### 4.3. Conclusions

Drawing on community pharmacists’ perspectives as professionals and frontline observers, this study demonstrates that antibiotic self-medication is perpetuated by structural barriers, entrenched misconceptions, and uneven regulatory enforcement, placing pharmacists at the centre of both the problem and potential solutions. To improve consultations and strengthen antimicrobial stewardship, pharmacists should implement structured, evidence-based counselling that directly addresses common misconceptions and supports informed decision-making [9,14,22,23]. Employing pictogram-enhanced or clearly written dosing instructions can improve comprehension and adherence, especially among patients with limited health literacy [29,31,32]. Brief, targeted myth-correction protocols during consultations may also promote appropriate antibiotic use [13,14]. Where feasible, integration of point-of-care diagnostic tools, such as C-reactive protein testing, can help to reduce unnecessary antibiotic recommendations and guide referrals appropriately [15,16,34]. Embedding these methods within consistent prescription-only enforcement and ongoing, public-facing education is most likely to achieve meaningful gains in stewardship [4,9,23].

## Figures and Tables

**Table 1:** Emergent Themes from Reflexive Thematic Analysis of 20 Interviews.

Theme	Unique Interviews	Total Mentions	Brief Description
Access and Self-Medication (Pharmacists’ frontline accounts)	19	45	Obtaining/using antibiotics without prescription; counter negotiation; leftover use; peer advice.
Relationship with Healthcare Professionals (Pharmacists’ professional roles)	19	39	Pharmacists’ educative role; referral to physicians; trust/pressure dynamics.
Knowledge and Beliefs about Antibiotics (Community narratives as recounted by pharmacists)	19	41	Misconceptions (e.g., treating viral illness), symptom heuristics, lay logics.
Use and Adherence to Treatment (Pharmacists’ observations of adherence behaviours)	16	34	Posology coaching; completing courses; dealing with side effects.
Healthcare System Barriers (Pharmacists’ lived constraints in practice)	20	47	Costs, queues, difficulty getting appointments/exams; system constraints fuel demand.
Regulation and Enforcement (Pharmacists’ experiences with regulatory application)	13	21	‘No sale without prescription’, local enforcement variability; pharmacists’ reliance on rules.

## Data Availability

Data available on request due to privacy restrictions.
